# Selective ERK1/2 agonists isolated from *Melia azedarach* with potent anti-leukemic activity

**DOI:** 10.1186/s12885-019-5914-8

**Published:** 2019-08-02

**Authors:** Ning Wang, Yanhua Fan, Chun-Mao Yuan, Jialei Song, Yao Yao, Wuling Liu, Babu Gajendran, Eldad Zacksenhaus, Yanmei Li, Jielin Liu, Xiao Jiang Hao, Yaacov Ben-David

**Affiliations:** 1 Department of Immunology State Key Laboratory for Functions and Applications of Medicinal Plants, Guizhou Medical University, Guiyang, 550025 China; 2 The Key Laboratory of Chemistry for Natural Products of Guizhou Province and Chinese Academic of Sciences, Baiyun District, Guiyang, 550014 China; 3 Department of Immunology, School of Basic Medical Sciences, Guizhou Medical University, Guiyang, 550025 China; 4 Department of Medicine, University of Toronto, Toronto, Ontario Canada; 5 Division of Advanced Diagnostics, Toronto General Research Institute, University Health Network, Toronto, Ontario Canada

**Keywords:** Cancer, Leukemia, Chinese medicinal plant, *Melia azedarach*, Drug screen, ERK1/2 agonists, Differentiation, Apoptosis

## Abstract

**Background:**

MAPK/ERK kinases transmit signals from many growth factors/kinase receptors during normal cell growth/differentiation, and their dysregulation is a hallmark of diverse types of cancers. A plethora of drugs were developed to block this kinase pathway for clinical application. With the exception of a recently identified agent, EQW, most of these inhibitors target upstream factors but not ERK1/2; no activator of ERK1/2 is currently available.

**Method:**

A library of compounds isolated from medicinal plants of China was screened for anti-cancer activities. Three limonoid compounds, termed A1541–43, originally isolated from the plant *Melia azedarach,* exhibiting strong anti-leukemic activity*.* The anti-neoplastic activity and the biological target of these compounds were explored using various methods, including western blotting, flow cytometry, molecular docking and animal model for leukemia.

**Results:**

Compounds A1541–43, exhibiting potent anti-leukemic activity, was shown to induce ERK1/2 phosphorylation. In contrast, the natural product Cedrelone, which shares structural similarities with A1541–43, functions as a potent inhibitor of ERK1/2. We provided evidence that A1541–43 and Cedrelone specifically target ERK1/2, but not the upstream MAPK/ERK pathway. Computational docking analysis predicts that compounds A1541–43 bind a region in ERK1/2 that is distinct from that to which Cedrelone and EQW bind. Interestingly, both A1541–43, which act as ERK1/2 agonists, and Cedrelone, which inhibit these kinases, exerted strong anti-proliferative activity against multiple leukemic cell lines, and induced robust apoptosis as well as erythroid and megakaryocytic differentiation in erythroleukemic cell lines. These compounds also suppressed tumor progression in a mouse model of erythroleukemia.

**Conclusions:**

This study identifies for the first time activators of ERK1/2 with therapeutic potential for the treatment of cancers driven by dysregulation of the MAPK/ERK pathway and possibly for other disorders.

**Electronic supplementary material:**

The online version of this article (10.1186/s12885-019-5914-8) contains supplementary material, which is available to authorized users.

## Background

The 5 years survival rate of cancer patients is gradually rising thanks to better detection and treatment methods [1]. Traditional treatments such as surgery, chemotherapy, and radiotherapy, together with advanced targeted therapy, immunotherapy and stem cell transplantation improved overall survival (OS) for many malignancies, yet, most patients, especially those with metastatic disease, succumb to their disease. Development of better targeted therapies is desperately needed to treat aggressive tumors.

Traditional Chinese medicine (TCM) was used for centuries in East Asia to cure diseases including cancer. Fruits and bark isolated from plant *Melia azedarach* were used in some TCMs to treat cancer [2, 3]. Recent studies also revealed antibacterial activity of *Melia azedarach* crude extract against streptococcus mutants [4]. In addition to cell cytotoxicity and antibacterial activity, extracts from *Melia azedarach* bark also display antioxidant activity [2–4]. Several limonoids including the well-known compound Cedrelone, isolated from this tree, display strong cytotoxic activities against several human cancer cell lines as well as bacterial species [5–9].

In this study, we isolated three compounds, structurally related to limonoids, from the *Melia azedarach* leaves. These compounds exhibited stronger in vitro toxicity against several leukemic cell lines than the structurally related agent Cedrelone. Strikingly, while Cedrelone inhibits ERK1/2, the three compounds we isolated exerted their toxicity by inducing ERK1/2. The compounds induced erythroid and megakaryocytic differentiation as well as apoptosis, and suppressed leukemogenesis in an animal model of leukemia. Both ERK1/2 agonists and the Cedrelone inhibitor then may have great potential for drug development for treatment of leukemias and other cancers with RAS/ERK activation.

## Methods

### Cell lines and proliferation analysis

Mycoplasma negative erythroleukemia cell lines CB3, CB7, K562 and HEL were all previously generated in our group or obtained from other laboratories, as described [10]. These cell lines were maintained in Dulbecco’s Modified Eagle Medium mixed with 5% fetal bovine serum (HyClone, GE Healthcare, Australia).

For IC50 determinations, cells (8 × 10^3^) were seeded in 96-well plates and treated with A1541, A1542, A1543 and Cedrelone at different concentrations for 3 days. Cells were then used in an MTT assay by adding 3-(4,5-dimethylthiazol-2-yl)-2,5-diphenyltetrazolium bromide to the culture for 4 h. Following removal of the supernatant, 200 ml DMSO was added to dissolve the formazan crystals. The absorbance was read using a Synergy2 modular Multi-Mode Reader (BioTek Instruments, Inc., Winooski, VT, USA) at 490 nm.

### In vivo leukemia induction and drug therapy

To induced erythroleukemia, One day born BALB/c mice (Male and females), bred in our pathgen free animal facility, were inoculated intraperitoneally (i.p.) by Friend Murine Leukemia Virus (F-MuLV), as described [10–12]. Five weeks post viral infection, mice were randomly grouped (*n* = 10) and injected i.p., every other day (Morning) for 2 weeks with A1542 and Cedrelone compounds (1 mg/kg) or control DMSO. After monitoring, mice displaying severe signs of late stage disease were sacrificed via cervical dislocation and used to calculate the % survival and spleen weight, as described elsewhere [10–12].

### Western blotting and inhibitory compounds

Western blotting was followed, was described elsewhere [12]. Polyclonal rabbit antibody for Fli-1 (ab133485), PKCδ (ab182126), Phospho- PKCδ (ab133456), MEK (ab178876), Phospho-MEK (ab96379), BAD (ab32445), and Phospho-BAD (ab129192) were obtained from Abcam (Abcam, Cambridge, UK); ERK (#4695) and phospho-ERK (#9101) from Cell Signalling Technology (CST, Danvers, MA01923, USA), β-actin (20536–1-AP) and GAPDH (13937–1-AP), from Proto-Technology (Protein-Tech, Bucuresti, Romania). Antibody dilution according to the manufacturer instructions.

The inhibitor of MEK (U0126) [#S1102] were obtained from Sellectchem (Sellectchem, Houston, USA) and PKC agonist Phorbol 12-myristate 13-acetate (TPA) from Sigma, (Sigma, St. Louis, MO, USA). These compounds were dissolved in DMSO and added to the cells at indicated concentration, as described in the results section.

### Flow cytometric analysis

Changes in the expression of various molecules in the compound-treated and control (DMSO-treated) cell lines were determined by flow cytometry, as described [12]. In brief, leukemic cells (10^6^) were first incubated with human Fc receptor binding inhibitor or CD16/CD32 blocking antibody (eBioscience, San Diego, CA, USA) for 20 min. Cells were then stained with the following primary antibodies for 1 h on ice. Primary antibodies were: Allophycocyanin-conjugated anti-mouse CD71 and Phycoerythrin-conjugated anti–mouse or anti-human CD41, CD61, Ter119, CD235a (eBioscience). After washing the cells with Phosphate Buffered Saline (PBS), a minimum of 10000 events were collected using the FACSCalibur flow cytometer (BD Biosciences, Beijing, China) and analyzed using CellQuest Pro software (BD Biosciences).

Antibodies for Flow cytometer analysis were obtained as follows; CD16 (#560248), CD41 (#555468), CD61 (#555753), Ter119 (#557915), CD235a (#555569), CD71 (#555534) and Annexin V (#550475) from Becton Dickinson (BD) (BD, Salt Lake City, Utah, USA), CD32 (#740399) and PI (#745153) from BD Optibuild (BD Biosciences, San Jose, CA, USA).

### Apoptosis and cell cycle analysis

To measure the effect of compounds on apoptosis and cell cycle, cells were incubated with the agents or DMSO for 24 h and then washed by cold PBS. For measure apoptosis, cells (10 ^6^) were stained with Annexin V and PI apoptosis detection Kit (BD Biosciences, Franklin lakes, NJ. Cat#556570), following the kit guidelines and analyzed by flow cytometer. For cell cycle analysis, drug treated and control cells (10^6^) were first fixed by cold 75% ethanol overnight at − 20 °C, washed once with cold PBS, stained in PI for 40 mins at 37 °C and then analyzed by flow cytometer, as described above.

### Animal experiments and statistics

Survival rates of mice treated with the compounds and DMSO were calculated and plotted using the nonparametric Kaplan-Meier analysis, as described [12]. Statistical analysis in all experiments was performed using the two-tailed Student t-test and significance determined by analysis of variance using Origin 3.5 software (Microcal Software, Northampton, MA, USA). *P* < 0.005 by **.

### Docking analysis

Molecular docking analysis was performed to demonstrate the 3-dimensional structures of A1541–43 and their target ERK and the results were drawn in chen sketch. The Crystallographic structure of ERK protein (PDB ID: 5JVT) was obtained from www.rcsb.org. Auto Dock tools 1.5.6. was used to extract molecular docking simulations, as described [13].

## Results

### Effect of new Limonoid compounds A1541, A1542 and A1543 on erythroleukemia cell lines in culture

**Fig. 1:**
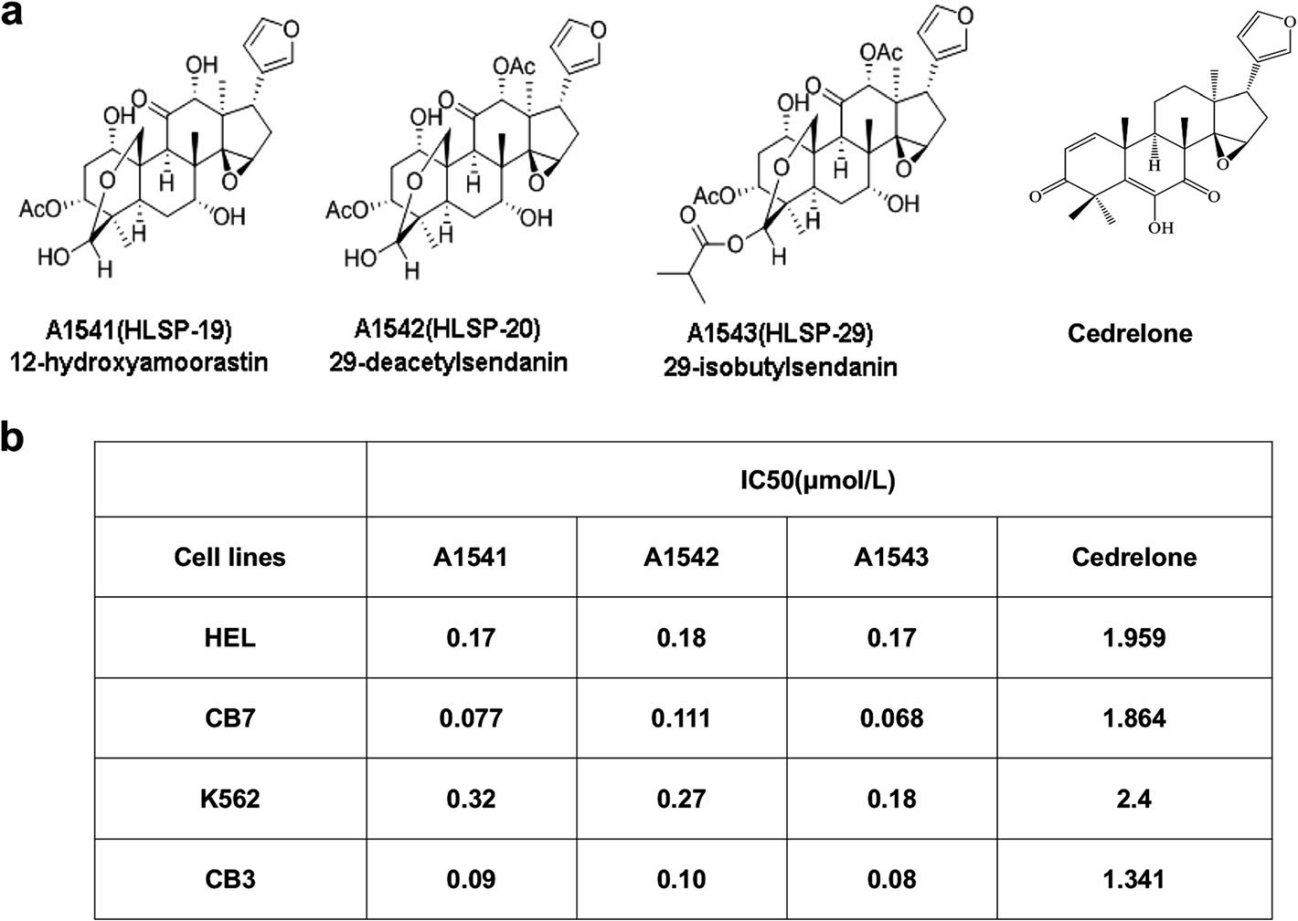
*Structure and biological activity of compounds A1541, A1542 and A1543*. **a** Chemical structure of A1541/A1542/A1543 and their related compound Cedrelone. **b** The anti-cancer activity of the compounds on the indicated cell lines measured by IC50

Compounds A1541/HLSP-19 (12-hydroxyamoorasti), A1542/HLSP-20 (1-deacetylsendani) and A1543/HLSP29 (29-iso-butylsendanin) were isolated from leaves’ extract from the *Melia azedarach* plant, as previously described [14]. These three compounds have similar structures to Limonoid’s Cedrelone, a known anti-cancer agent [6, 7] (Fig. 1a). The inhibitory effect of A1541–43 compounds was compared with Cedrelone using several human and mouse erythroleukemic cell lines by MTT assays. These revealed 9–20 fold reduced IC50 of compounds A1541–43 versus Cedrelone (Fig. 1b). Dose dependent inhibitory effect of all 4 compounds on mouse and human erythroleukemia cell lines CB7 and HEL is shown in Additional file 1: Figure S1A and Additional file 1: Figure S2A. While CB7 cells treated with 0.5 μM of A1541–43 died 24 h post drug treatment (Additional file 1: Figure S1B), HEL treated cells survived, but showed lower number of cells (Additional file 1: Figure S2B). Cedrelone displayed no effect on CB7 and HEL cells at 0.5 μM drug concentration, but killed most cells at 5 μM within 24 h of incubation (Additional file 1: Figure S1B and Additional file 1: Figure S2B). These drug inhibition patterns suggest that A1541/43 are either more potent than Cedrelone or employ different mechanisms to suppress leukemic cell proliferation.

### A1541–43 and Cedrelone induce cell cycle arrest and apoptosis in leukemic cells

Compounds A1541–43 and Cedrelone induced rapid apoptosis of CB7 cells 24 h post-drug treatment (Additional file 1: Figure S3A). A1541–43, but not Cedrelone, showed limited apoptosis of HEL cells (Additional file 1: Figure S3B), which is consistent with higher survival and growth inhibition in culture (Additional file 1: Figure S2B).

A1541–43 induced strong cell cycle arrest in both CB7 and HEL cells by increasing the percentage of cells in S phase (Additional file 1: Figure S4). Interestingly, Cedrelone also inhibited cell cycle, but at the G2 phase (Additional file 1: Figure S4). These results further suggest that A1541–43 use different mechanism than Cedrelone to block cell growth and proliferation.

### Effect of A1541–43 and Cedrelone on erythroleukemia differentiation

**Fig. 2:**
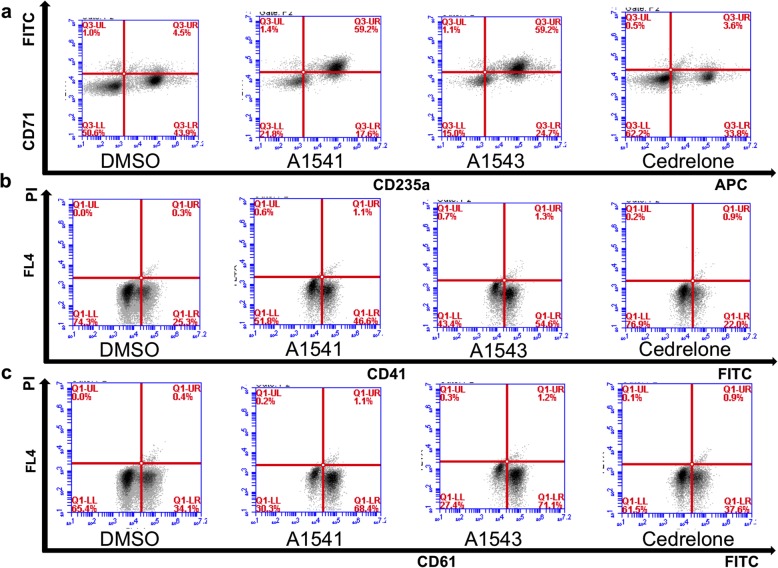
*Induction of megakaryocytic or erythroid differentiation in erythroleukemia cell line HEL by A1541 and A1543.* **a-c** Treatment of the erythroleukemic cell lines HEL with A1542–43 increases the percentage of CD71/CD235 positive erythroid cells (**a**), as well as CD41+ (**b**) and CD61+ (**c**) megakaryocytic cells after 24 h. Cedrelone had no effect on either erythroid or megakaryocytic differentiation (**a-c**)

**Fig. 3:**
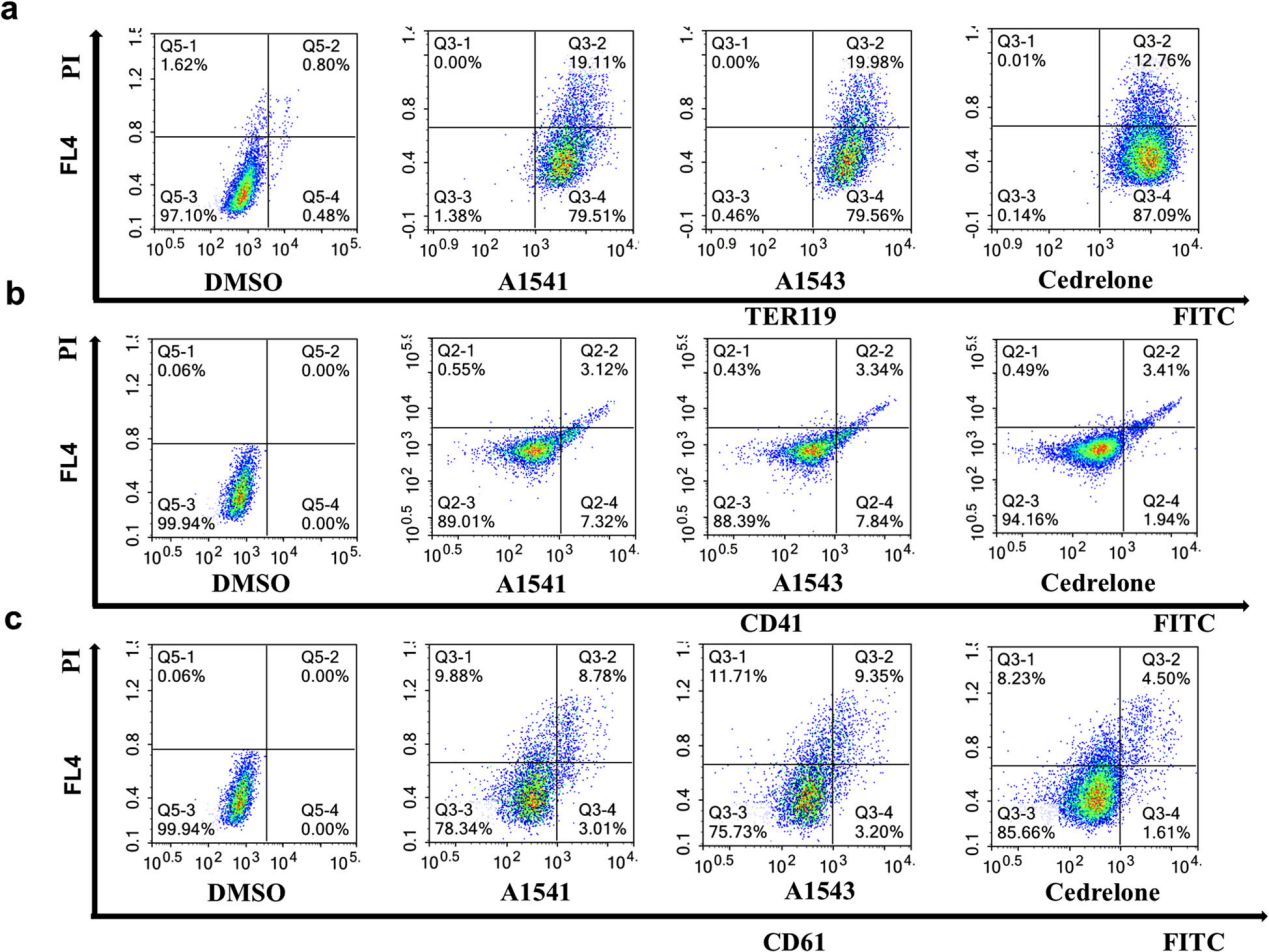
*Induction of megakaryocytic or erythroid differentiation in erythroleukemia cell line CB7 by A1541 and A1543.* **a-c** Treatment of the erythroleukemic cell lines CB7 with A1541 and A1543 increases the percentage of CD71/TER119+ erythroid cells (**a**), but has no effect on percentage of CD41+ (**b**) and CD61+ (**c**) megakaryocytic cells after 24 h. Cedrelone had no effect on erythroid and megakaryocytic differentiation of CB7 cells (**a**-**c**)

To determine the effect of the compounds on differentiation, expression of several erythroid (CD71/CD235) and megakaryocytic (CD41/CD61) differentiation markers was determined in murine erythroleukemia CB7 and human HEL cell lines after drug treatment. While A1541 and A1543 induced the erythroid marker CD71/CD235 in HEL cells, Cedrelone slightly downregulated this marker (Fig. 2a). However, in CB7 cells, A1541, A1543 and Cedrelone all significantly increased expression of the erythroid TER119 marker (Fig. 3a). A1542 also induced similar pattern of erythroid differentiation in both HEL and CB7 cells (not shown).

In HEL cells, expression of megakaryocytic markers CD41 and CD61 was induced following 3 days incubation with A1541 and A1543, but not with Cedrelone (Fig. 2b-c). Longer incubation of HEL cells with A1542 did not further increase this level of differentiation (data not shown). In CB7 cells, none of these compounds induced CD41/CD61 expression (Fig. 3b-c). These results confirm our previous observation that HEL progenitor cells can differentiate to megakaryocytic or erythroid lineages whereas CB7 cells are restricted to erythroid differentiation [12]. Thus, both A1541/43 and Cedrelone can induce erythroid and/or megakaryocytic differentiation of erythroleukemic cells.

### The A1541-A1543 compounds specifically activate the ERK1/2 kinase in leukemic cells

**Fig. 4:**
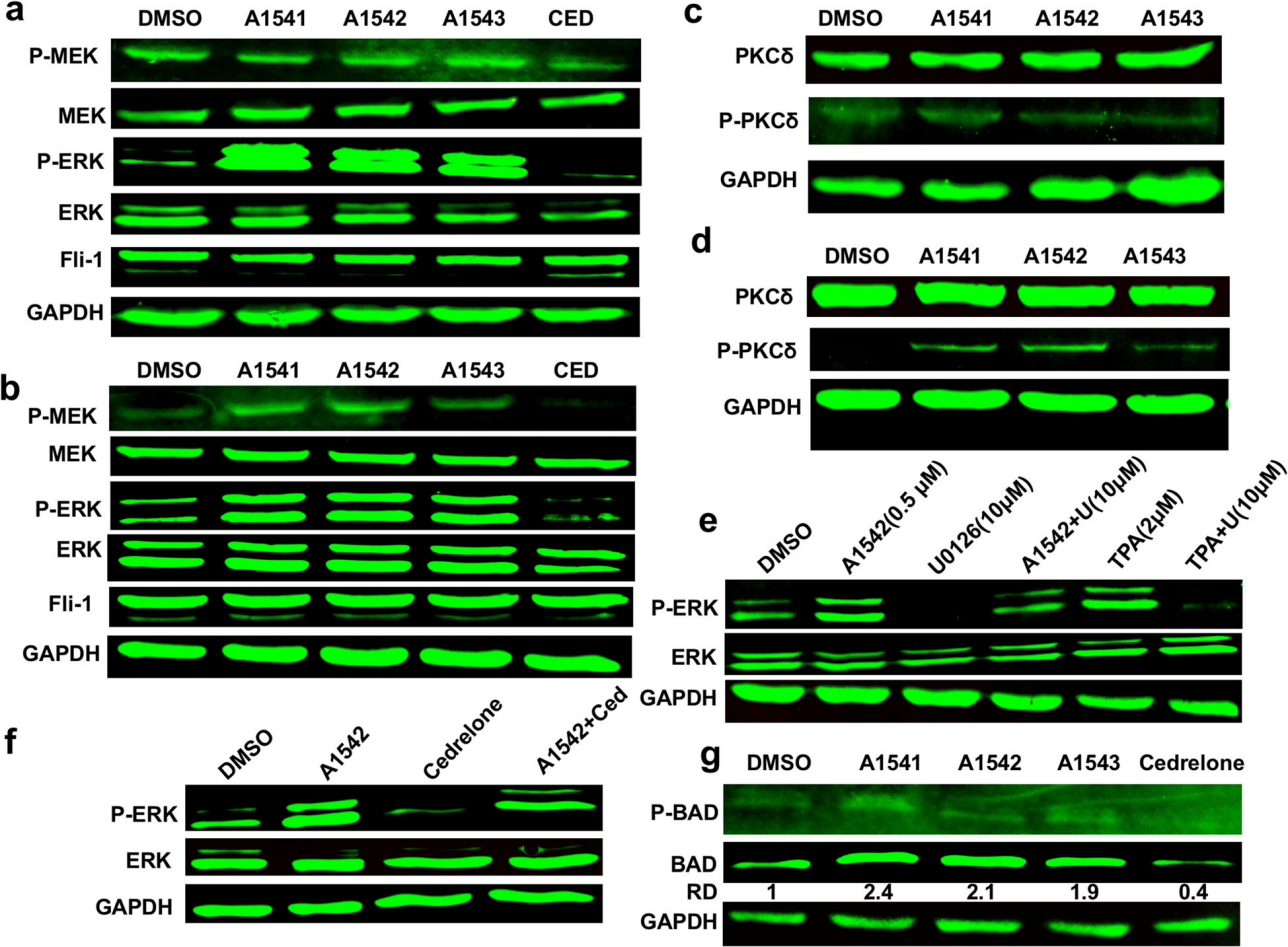
*A1541, A1542 and A1543 alter phosphorylation of ERK1/2.* **a** and **b** A1541–43 (0.5uM) induce phosphorylation of MAPK/ERK in HEL (**a**) and CB7 (**b**) cells, 24 h post-drug treatment. Upstream MEK phosphorylation was only slightly induced by these compounds in CB7 (**b**) and not at all in HEL (**a**) cells. **c** and **d** A1541–43 did not alter phosphorylation of PKCδ in HEL cells (**c**), but slightly increased PKCδ phosphorylation in CB7 cells (**d**). **e** Induction of MAPK/ERK by A1542 is not blocked by the MEK inhibitor U0126 (U), after treatment of HEL cells for 24 h with the indicated drug concentrations. However, U0126 blocksed TPA-induced phosphorylation of ERK1/2. **f** In HEL cells, Cedrelone (5uM) significantly reduced ERK1/2 phosphorylation induced by A1542 (0.5uM), after 24 h of drug treatment. **g** The compounds A1541-43 were significantly increased the expression of active unphosphorylated form of BAD, but Cedrelone in contrast reduced  its expression. Relative Density (RD)

To uncover mechanisms of growth inhibition by these new compounds, we determined their effect on growth and differentiation signaling pathways including MAPK/ERK activity. Remarkably, while Cedrelone suppressed MAPK/ERK phosphorylation relative to DMSO control, A1541–43 strongly activated MAPK/ERK phosphorylation in both HEL (Fig. 4a) and CB7 (Fig. 4b). Interestingly, A1541–43 did not significantly increase the phosphorylation of MEK, the upstream kinase of MAPK/ERK (Fig. 4a-b) or PKCδ [12] in HEL (Fig. 4c) and CB7 (Fig. 4d) cells. This observation suggests that A1541–43 act as specific activators (agonists) of ERK1/2. To corroborate this conjecture, we treated HEL cells with A1542 in the presence or absence of the MEK inhibitor U0126 (10uM) [12]. While U0126 completely reduced ERK1/2 phosphorylation relative to DMSO treated cells, this inhibitor only partially reduced phosphorylation of this kinase in the presence A1542 (Fig. 4e). In contrast, U0126 completely suppressed ERK phosphorylation induced by TPA, a potent activator of PKCδ [12]. In HEL cells Cedrelone did not affect MEK phosphorylation (Fig. 4a), but partially blocked the induction of ERK1/2 phosphorylation by A1542 (Fig. 4f). The pro-apoptotic factor BAD is inhibited by phosphorylation on Ser112 by various survival factors including ERK [15, 16]. In line with the idea that A1541-A1543 act as agonists of ERK, we found that A1542 significantly induced total BAD expression (Fig. 4g). Notably, the level of inactive Ser112-phospho-Bad expression was very low and only detectable after long exposure. A1542-induced ERK1/2 likely have additional targets, yet to be identified, that promote apoptosis to overcome the effect on BAD.

**Fig. 5:**
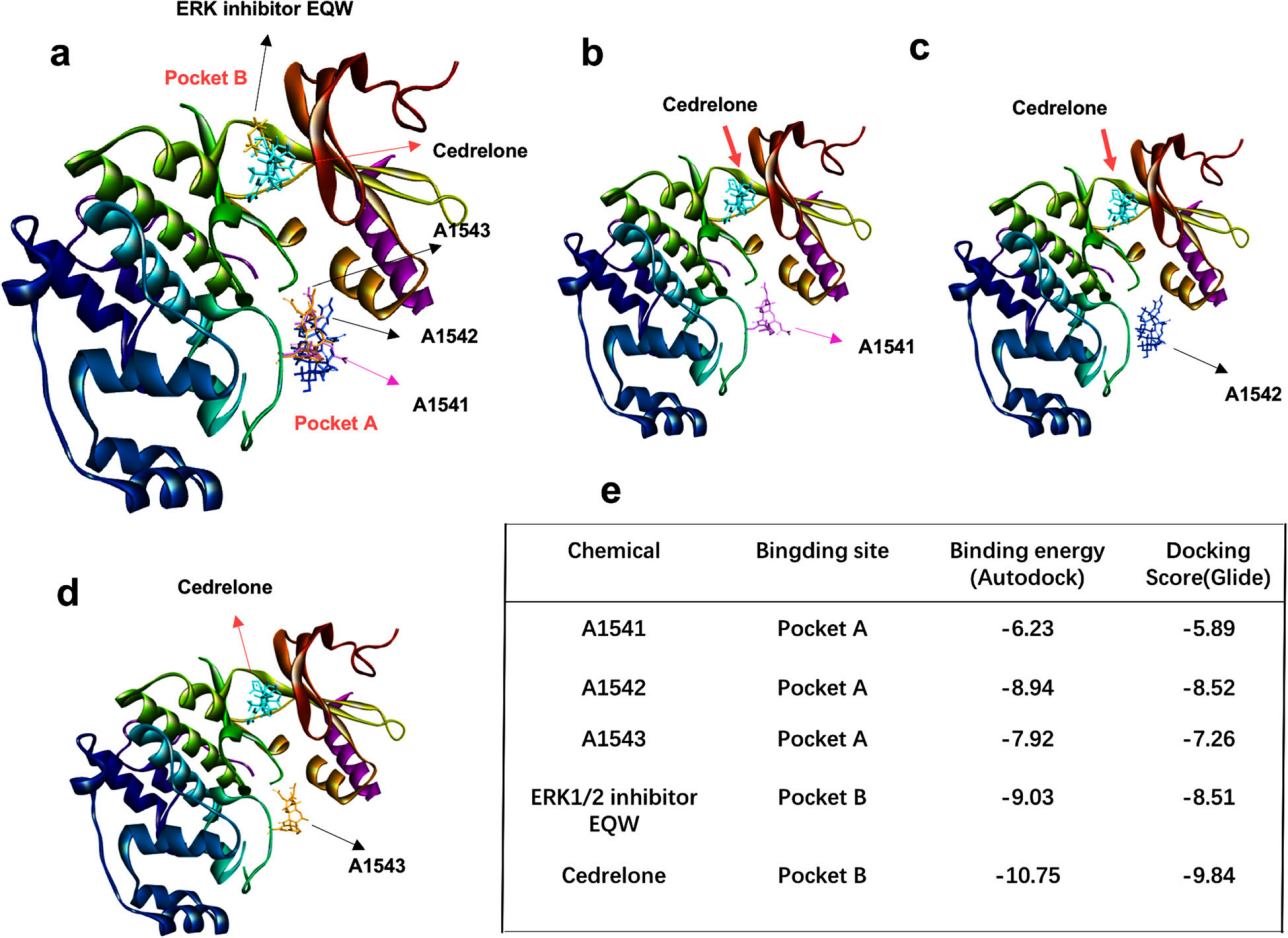
*A1541–43 are predicted to bind a pocket within the ERK1/2 protein.* **a** Binding of A1541–43, EQW and Cedrelone within pocket A and B of ERK1/2. **b** Relative binding of Cedrelone and A1541 (**b**), Cedrelone and A1542 (**c**), Cedrelone and A1543 (**d**) within the pocket B and A of ERK1/2, respectively. **e** Properties of drug binding affinities to ERK1.2

We next employed computational docking analysis to determine if the A1541-A1543 compounds are predicted to interact directly with ERK1/2 vis-à-vis Cedrelone. We performed computational docking analysis of A1541-A1543 compounds against the ERK1/2 protein. A recent study has shown that the ERK1/2 specific inhibitor EQW **(**5-chloranyl-~[12]-(oxan-4-yl)pyrimidin-2-amine, PDB# 6G91) binds pocket B of the kinase [17]. Interestingly, like EQW, Cedrelone was found to occupy pocket B (Fig. 5a). In striking contrast, A1541–43 were predicted to bind the nearby pocket A domain 2, a property consistent with their ability to activate ERK1/2 (Fig. 5a-d). Cedrelone and EQW interacted to pocket B with predicted affinity of 9.03 and 10.75, respectively (Fig. 5e). In contrast A1541–43 bound pocket A with affinity ranging from 6.23–8.95, with A1542 having higher binding affinity as compared to A1541 and A1543 (Fig. 5e). The interaction of the five compounds with amino-acids and relative position in ERK1/2 are shown in Additional file 1: Figure S5.

### Effect of A1541-A1543 on leukemia progression

**Fig. 6:**
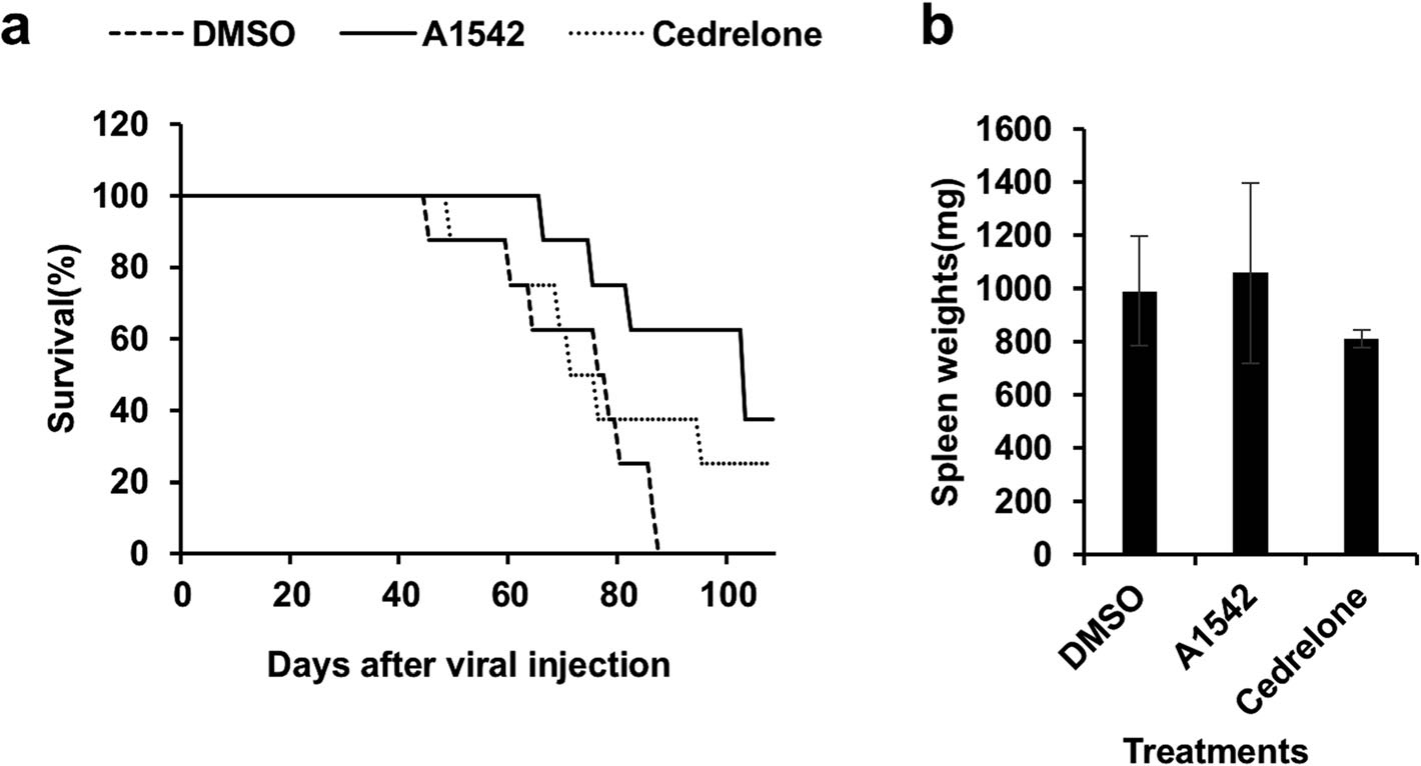
*Inhibition of leukemogenesis by A1542 and Cedrelone.* **a** Newborn BALB/c mice (*n* = 10 per group) were infected with F-MuLV and 6 weeks later treated with A1542 or Cedrelone (1 mg/kg) every other day for a total of six injections. Latency to death was used to plot a Kaplan-Meier survival curve [12]. **b** Spleen weights of leukemic mice were measure at endpoint. Experiment repeated 3 times

We next examined effect of our compounds on leukemia progression using a mouse model of erythroleukemia, induced by a single dose of oncogenic virus, as described [10, 11, 18]. New born BALB/c mice were injected with friend erythroleukemia virus and 6 weeks later, when tumors are induced due to retroviral insertional activation of Fli-1 [19], leukemic mice (*n* = 10) were treated with A1542, Cedrelone (1 mg/kg) and DMSO, every other day for a total of 6 injections. The drug treated mice were then monitored for leukemia development. Compound A1542 exerted a stronger anti-leukemic activity than Cedrelone, compared with control DMSO-treated group (Fig. 6a). No statistically significant changes in spleen size or sites of tumor infiltration were seen at time of death between mice treated with A1542, Cedrelone or DMSO (Fig. 6b). These results indicate that both inhibition and activation of ERK1/2 can suppress leukemic progression.

## Discussion

This study report of the discovery of three compounds A1541–43, isolated from Chinese medicinal plant Melia, which act as potent agonists of ERK1/2. These compounds display a stronger inhibitory effect on leukemic cells than their structurally related compound Cedrelone, which acts as ERK1/2 antagonist. Computerized docking analysis reveals high affinity binding of A1541–43 to a region of ERK, which is distinct from the nearby region to which Cedrelone binds. Increased ERK1/2 activation by A1541–43 resulted in induced erythroid and megakaryocytic differentiation as well as apoptosis; ERK inhibition by Cedrelone induced erythroid differentiation and cell death. Our observation that both A1542 and Cedrelone suppressed leukemogenesis in a mouse model demonstrate that both activation and inactivation of ERK1/2 is beneficial for treatment of leukemia and possibly other malignancies.

Many compounds targeting the MAPK/ERK pathway have been identified and some show promising preclinical activity [20]. These inhibitory compounds target upstream regulators of the MAPK/ERK pathway, including BRAF and MEK kinases, but not ERK1/2 or downstream factors [20]. Clinical use of these upstream inhibitors often results in drug resistance through amplification of the ERK1/2 gene [21, 22]. To overcome this, Heightman TD et al., recently used a fragment-based library to identify the ERK1/2 inhibitor EQW [17]. While the anti-cancer activity of EQW has not yet been determined, ERK1/2 inhibitor Cedrelone identified in this study binds to similar binding pocket in ERK1/2 as does EQW, resulting in inhibition of the ERK1/2 phosphorylation. In contrast, A1541–43 interact with a different pocket domain in ERK1/2, causing over-phosphorylation and activation, which also induces strong suppression of leukemic cell growth. Thus, our study for the first time identities novel activators and an inhibitor of ERK1/2 with immediate application for treatment of leukemia.

Previous studies have shown that ERK can promote proliferation but also induce cell death depending on context [23]. While the mechanisms by which A1541–43 induce differentiation and apoptosis through induction of ERK1/2 phosphorylation remain to be elucidated, we showed herein that they induced the active form of unphosphorylated BAD. Multiple additional targets of A1541–43 and phosphorylated ERK1/2, yet to be identified, likely promote its anti-proliferative activity on leukemia, as shown here, and on other liquid and solid tumors (unpublished data).

Both ERK1/2 agonists and Cedrelone were shown to induce either erythroid or megakaryocytic differentiation or both. In human, many mutations are associated with inherited or acquired deficiency in either erythroid, megakaryocyte or both [24]. These compounds are therefore potential candidate for treatment of these disorders. Indeed, these compounds may have advantage over drugs targeting upstream MAPK signaling pathway as they may control less downstream effectors, causing less side effects and toxicity.

## Conclusion

This study classifies the natural product Cedrelone as an ERK inhibitor and for the first time identifies three chemically related compounds, A1541–3, as ERK1/2 agonists. Both ERK activation and inhibition were shown to have anti-leukemic activity demonstrating tumor cell sensitivity to alterations in activity of these kinases. These ERK1/2 inhibitory/agonistic compounds are therefore strong candidate for future drug development.

## Additional file

Additional file 1: **Figure S1.** Growth inhibition of HEL cells by the compounds. (**A**) HEL cells were treated with the indicated concentration of the compounds and percentage of inhibition was determined by MTT assay 3 days after drug treatment. (**B**) Microscopic images of the cells after 2 days treatment with the indicated compounds (magnification × 40). *P* < 0.005 denoted by **. **Figure S2.** **G**rowth inhibition of CB3 cells by the compounds. (**A**) HEL cells were treated with the indicated concentration of the compounds and percentage of inhibition was determined by MTT assay 3 days after drug treatment. (**B**) Microscopic images of the cells after 2 days treatment with the indicated compounds (magnification × 40). *P* < 0.005 denoted by **.Scale bar: 20 μm. **Figure S3.** Induction of Apoptosis by the compounds in erythroleukemic cells. (**A**) A1541–43 and Cedrelone treatment increased the percentage of Annexin V-positive apoptotic cells in CB7 cells 24 h post drug treatment. (**B**) While A1541–43 compounds failed to increase the percentage of apoptosis in HEL cells, Cedrelone was able to promote cell death in these cells. **Figure S4.** Induction od cell cycle arrest by the compoundss in erythroleukemic cells. In HEL (**A**) and CB7 (**B**) cells, A1541/43 increase S1 and decrease G1 and G2 phase stages of cell cycle, 24 h post-drug incubation. Cedrelone in contract increases G2 and decreases G1 and S in both cell lines (A and B G1 and G2 phase stages of cell cycle, 24 h post-drug incubation. Cedrelone in contract increases G2 and decreases G1 and S in both cell lines (A and B). **Figure S5.** Chemical interaction of the compounds to the indicated ERK1/2 amino-acids. (PPT 6517 kb)

## Data Availability

All data and materials are available without restriction. Researchers can obtain data by contacting the corresponding authors.

## Abbreviations

DMSO: Dimethyl sulfate
ERK: Extracellular signal-regulated kinase
F-MuLV: Friend Murine Leukemia Virus (F-MuLV)
MAPK: Mitogen-Activated Protein Kinase
PKC: Protein Kinase C
TCM: Tradition Chinese Medicine
