# Supplementary material for: Selective ERK1/2 agonists isolated from Melia azedarach with potent anti-leukemic activity
Source: BMC Cancer. 2019 Aug 2;19:764. doi: 10.1186/s12885-019-5914-8 (PMC6679490; doi:10.1186/s12885-019-5914-8)
Supplement: Supplementary file 1 — Figure S1. Growth inhibition of HEL cells by the compounds. (A) HEL cells were treated with the indicated concentration of the compounds and percentage of inhibition was determined by MTT assay 3 days after drug treatment. (B) Microscopic images of the cells after 2 days treatment with the indicated compounds (magnification × 40). P < 0.005 denoted by **. Figure S2. Growth inhibition of CB3 cells by the compounds. (A) HEL cells were treated with the indicated concentration of the compounds and percentage of inhibition was determined by MTT assay 3 days after drug treatment. (B) Microscopic images of the cells after 2 days treatment with the indicated compounds (magnification × 40). P < 0.005 denoted by **.Scale bar: 20 μm. Figure S3. Induction of Apoptosis by the compounds in erythroleukemic cells. (A) A1541–43 and Cedrelone treatment increased the percentage of Annexin V-positive apoptotic cells in CB7 cells 24 h post drug treatment. (B) While A1541–43 compounds failed to increase the percentage of apoptosis in HEL cells, Cedrelone was able to promote cell death in these cells. Figure S4. Induction od cell cycle arrest by the compoundss in erythroleukemic cells. In HEL (A) and CB7 (B) cells, A1541/43 increase S1 and decrease G1 and G2 phase stages of cell cycle, 24 h post-drug incubation. Cedrelone in contract increases G2 and decreases G1 and S in both cell lines (A and B G1 and G2 phase stages of cell cycle, 24 h post-drug incubation. Cedrelone in contract increases G2 and decreases G1 and S in both cell lines (A and B). Figure S5. Chemical interaction of the compounds to the indicated ERK1/2 amino-acids. (PPT 6517 kb) [file 12885_2019_5914_MOESM1_ESM.ppt]

## Slide 1
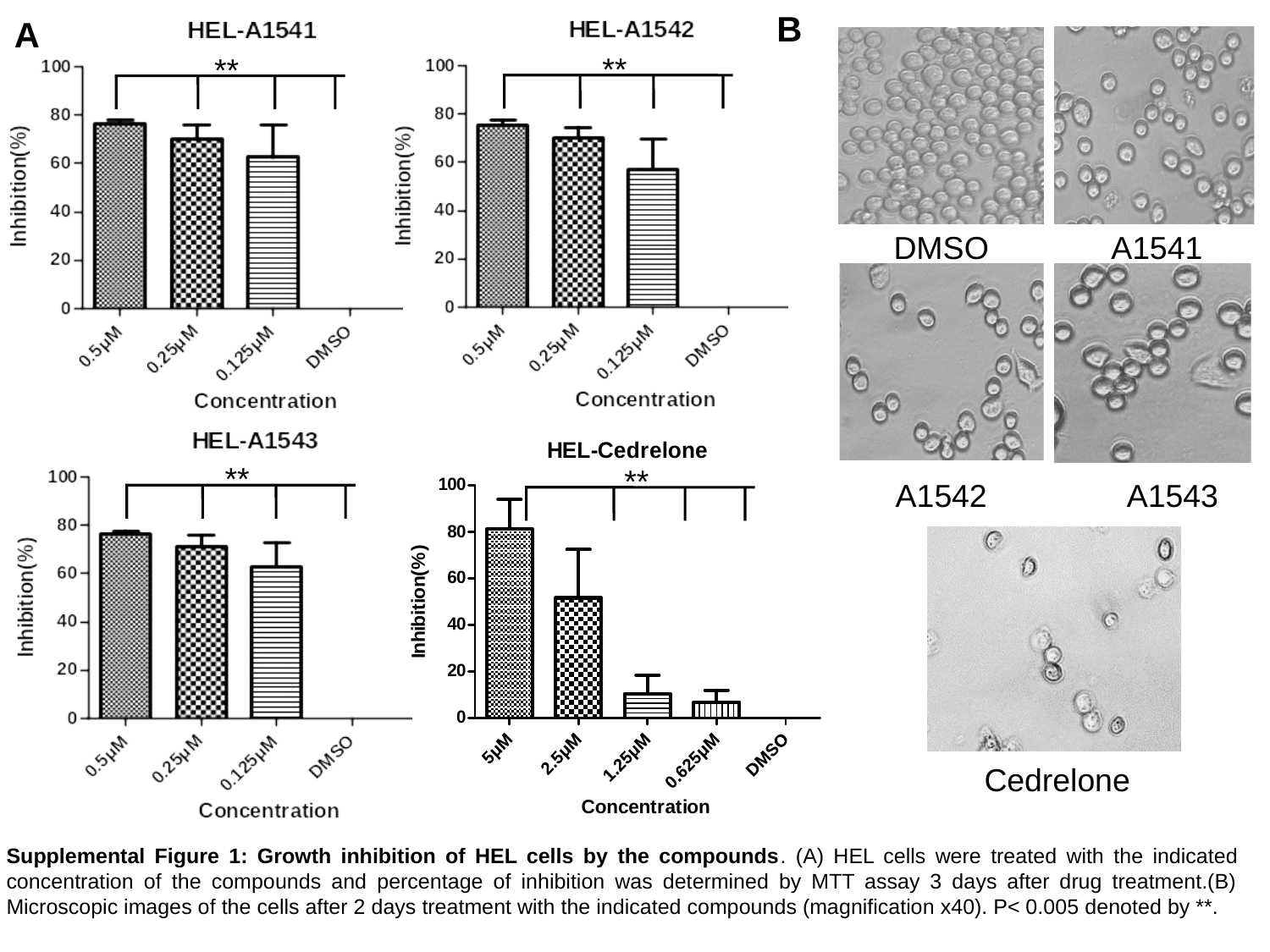

B
A
DMSO A1541
A1542 A1543
Cedrelone
**
**
**
**
Supplemental Figure 1: Growth inhibition of HEL cells by the compounds. (A) HEL cells were treated with the indicated concentration of the compounds and percentage of inhibition was determined by MTT assay 3 days after drug treatment.(B) Microscopic images of the cells after 2 days treatment with the indicated compounds (magnification x40). P< 0.005 denoted by **.

## Slide 2
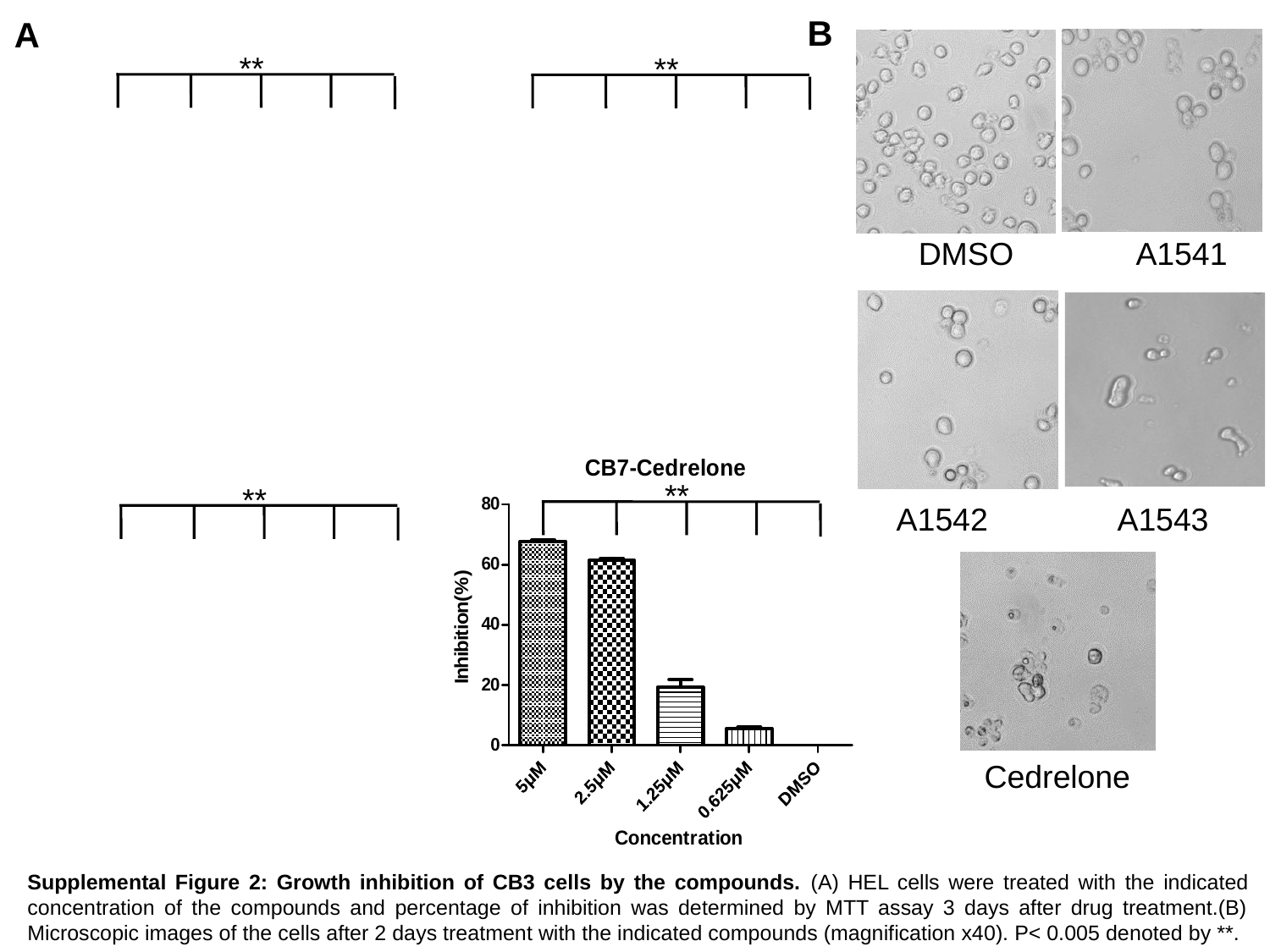

B
A
DMSO A1541
A1542
A1543
Cedrelone
**
**
**
**
Supplemental Figure 2: Growth inhibition of CB3 cells by the compounds. (A) HEL cells were treated with the indicated concentration of the compounds and percentage of inhibition was determined by MTT assay 3 days after drug treatment.(B) Microscopic images of the cells after 2 days treatment with the indicated compounds (magnification x40). P< 0.005 denoted by **.

## Slide 3
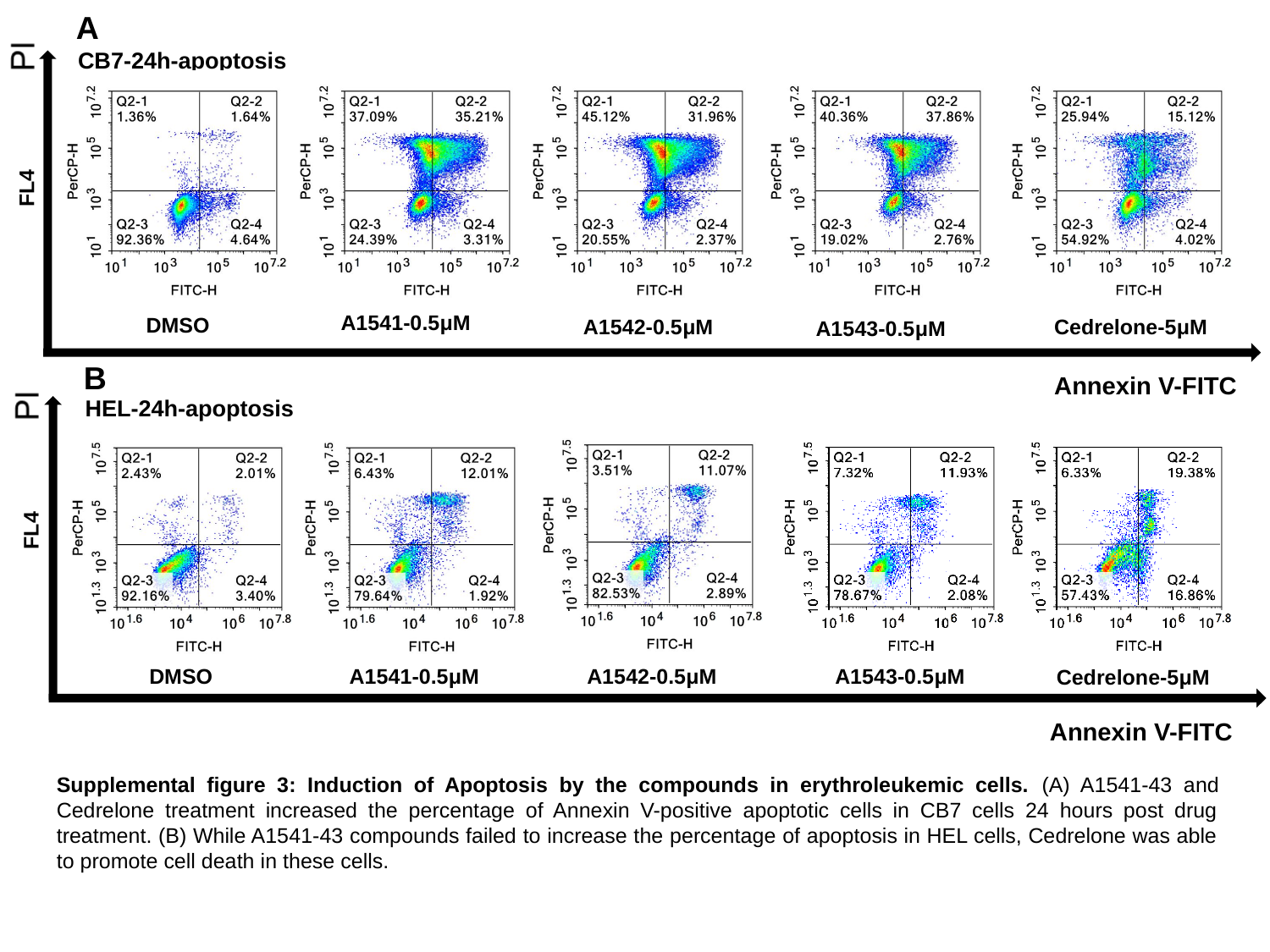

A
CB7-24h-apoptosis
A1541-0.5μM
DMSO
A1542-0.5μM
A1543-0.5μM
Cedrelone-5μM
Annexin V-FITC
FL4
B
HEL-24h-apoptosis
A1542-0.5μM
DMSO
A1541-0.5μM
A1543-0.5μM
Cedrelone-5μM
Annexin V-FITC
FL4
Supplemental figure 3: Induction of Apoptosis by the compounds in erythroleukemic cells. (A) A1541-43 and Cedrelone treatment increased the percentage of Annexin V-positive apoptotic cells in CB7 cells 24 hours post drug treatment. (B) While A1541-43 compounds failed to increase the percentage of apoptosis in HEL cells, Cedrelone was able to promote cell death in these cells.

## Slide 4
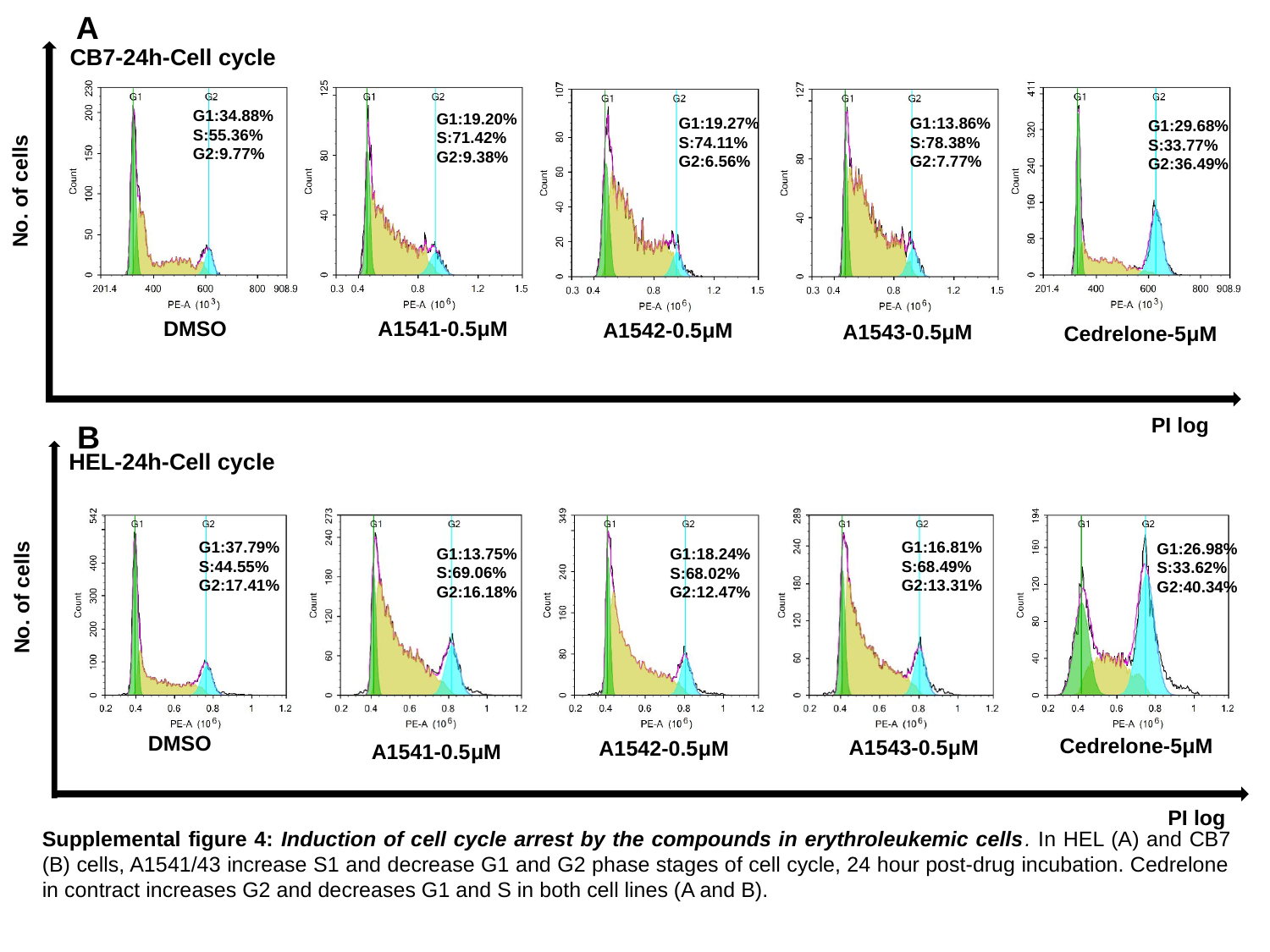

A
CB7-24h-Cell cycle
G1:34.88%
S:55.36%
G2:9.77%
G1:29.68%
S:33.77%
G2:36.49%
DMSO
A1541-0.5μM
A1542-0.5μM
A1543-0.5μM
Cedrelone-5μM
G1:19.20%
S:71.42%
G2:9.38%
G1:13.86%
S:78.38%
G2:7.77%
G1:19.27%
S:74.11%
G2:6.56%
No. of cells
PI log
B
HEL-24h-Cell cycle
G1:37.79%
S:44.55%
G2:17.41%
G1:13.75%
S:69.06%
G2:16.18%
G1:18.24%
S:68.02%
G2:12.47%
G1:16.81%
S:68.49%
G2:13.31%
G1:26.98%
S:33.62%
G2:40.34%
DMSO
A1541-0.5μM
A1542-0.5μM
A1543-0.5μM
Cedrelone-5μM
No. of cells
PI log
Supplemental figure 4: Induction of cell cycle arrest by the compounds in erythroleukemic cells. In HEL (A) and CB7 (B) cells, A1541/43 increase S1 and decrease G1 and G2 phase stages of cell cycle, 24 hour post-drug incubation. Cedrelone in contract increases G2 and decreases G1 and S in both cell lines (A and B).

## Slide 5
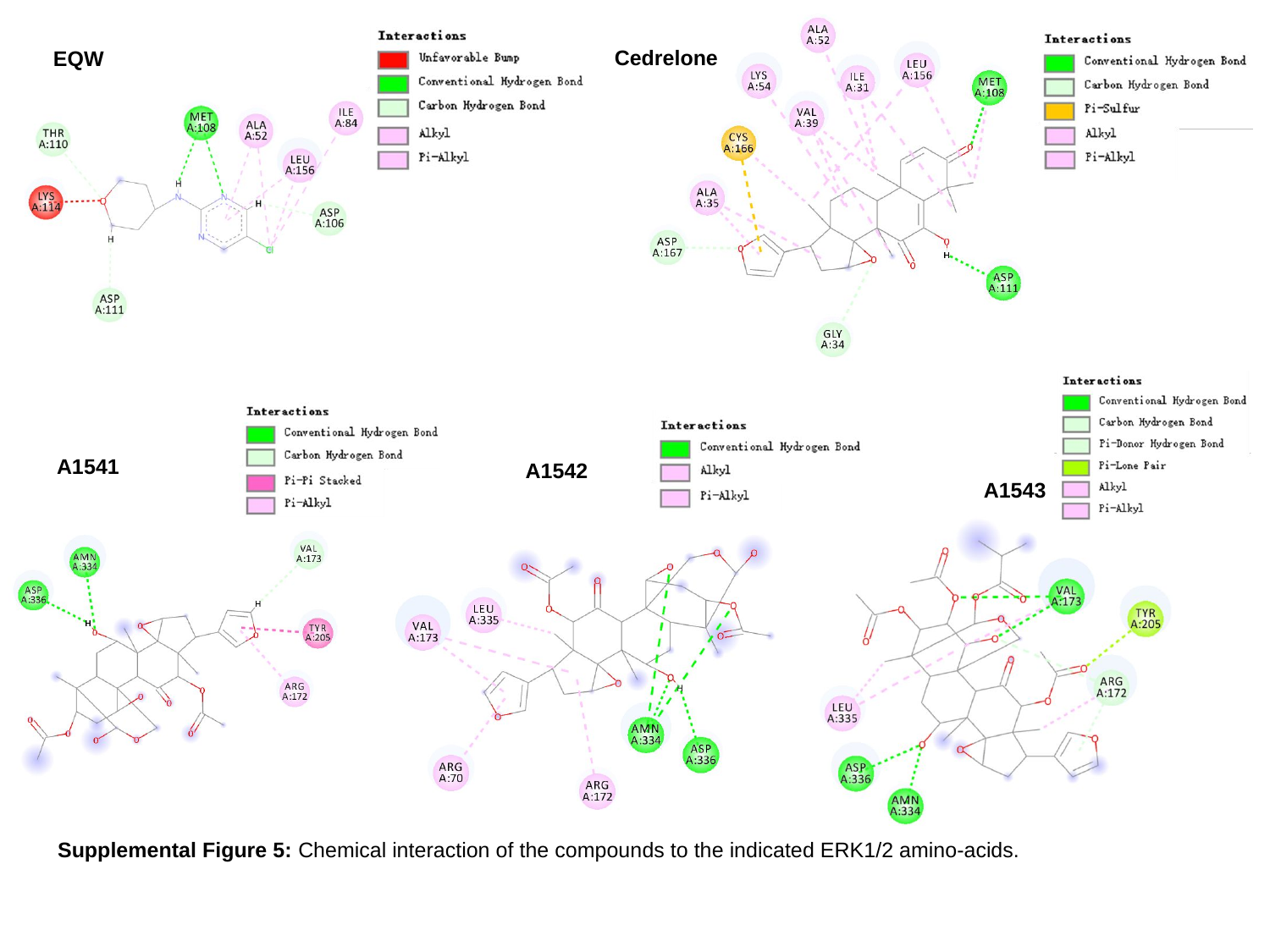

Cedrelone
EQW
A1541
A1542
A1543
Supplemental Figure 5: Chemical interaction of the compounds to the indicated ERK1/2 amino-acids.
